# Regional Geriatric Trauma Advice for Older Patients with Chronic Subdural Haematoma—A Novel Service for Multi-Disciplinary Decision-Making

**DOI:** 10.3390/geriatrics11040097

**Published:** 2026-08-03

**Authors:** Frances Rickard, Rebecca Hutchins, David Shipway, Adam Williams, Anthony Cox, Alex Mortimer

**Affiliations:** Southmead Hospital, Bristol NHS Foundation Trust, Bristol BS10 5NB, UK

**Keywords:** chronic subdural haematoma, older neurosurgical patients, frailty, multidisciplinary care

## Abstract

**Background:** Chronic subdural haematoma (cSDH) is one of the most common neurocranial conditions in older adults and is increasing in incidence. Conservatively managed patients are typically older, frailer, and more comorbid than surgical candidates, yet specialist geriatrician input into their management is rarely formalised. Potential for geographic inequity in hub-and-spoke neurosurgical networks within the UK compounds this challenge. We describe a service evaluation of a novel regional geriatric trauma advice service providing specialist trauma geriatrician input into cSDH management alongside the neurosurgical team, across a single UK Neurosurgical Network. To the authors’ knowledge, this is the first of its kind in the UK. Prior to this service ‘normal’ care within our region was no routine specialist medical input. **Methods:** A service evaluation of all referrals received by the North Bristol National Health Service (NHS) Trust geriatric trauma team between July 2024 and March 2026 was performed by the authors. Referrals were discussed within a multidisciplinary team including geriatric trauma, interventional neuroradiology and neurosurgery. Individuals’ demographic data, advice types, management enhancement, intervention outcomes and mortality were recorded and analysed descriptively. Management enhancement was defined as a definitive management decision that resulted in either an action or an active decision not to act. **Results:** Ninety referrals were included. Median age was 83 years (IQR 77–88) and median Clinical Frailty Scale (CFS) score was 4, with 50% classified as frail (CFS 4–8). Referrals were received from five satellite NHS trusts and the community. Three-quarters (75.6%) required multiple concurrent advice types; Middle Meningeal Artery Embolisation (MMAE) suitability (93.3%) and antithrombotic management (51.1%) were the most frequent. Specialist geriatrician input enhanced management in 97.8% of cases. Forty percent of conservatively managed patients were converted to active intervention, most commonly standalone MMAE (50%). Of district general hospital patient referrals, 40.3% were conveyed to the neurosurgical centre following geriatric trauma review. Mortality was comparatively lower at multiple time points when compared to existing UK data for conservatively managed patients. **Conclusions:** A regional virtual geriatric trauma advice service for older adults with cSDH is feasible and is associated with enhanced management of this cohort, potentially improving access to treatment. Combining neurosurgical, neurointerventional and geriatrician expertise through a structured multi-disciplinary team (MDT) approach addresses the clinical complexity of this patient group and the potential geographic inequity of hub-and-spoke networks. Further research is needed to investigate the impact of routine geriatrician input into cSDH care.

## 1. Introduction

Chronic subdural haematoma (cSDH) is one of the most common neurocranial conditions in older adults [1], with an overall incidence of 1.7–20.6 per 100,000 persons per year [2]. This already costs the UK around £42 million annually and is expected to rise to £79 million by 2040 [3]. The incidence of cSDH increases with age [4] and with that often comes multimorbidity, frailty, and antithrombotic use. Both frailty and multimorbidity have been linked to higher mortality and complication rates in cSDH [5,6] and the importance of frailty in understanding prognosis and outcomes in conservatively managed cSDH has been highlighted [7]. This supports the need for early geriatrician input in cSDH to help address this complex patient cohort [7].

A further challenge in cSDH management is the potential for geographic inequity. In the UK, neurosurgical care is based on a hub-and-spoke model, with a central tertiary neurosurgical ‘hub’, and surrounding regional district general hospital (DGH) ‘spokes’. In the Southwest, North Bristol NHS Trust (NBT) is the tertiary centre for the Severn neurosurgical network, providing adult neurosurgical care for Gloucestershire, Somerset and Wiltshire and the city of Bristol. The communities this network serves account for more than one million people with a variety of socio-economic conditions that come with differing health inequalities; for example the city of Bristol has a notably younger population with a higher degree of deprivation whereas North Somerset has an older population which comes with its own set of challenges [8]. The service is accessed from all other regional sites via the online referral system (Referapatient.org). Any patients not requiring urgent neurosurgical intervention, and hence hospital transfer, are managed at spoke centres with neurosurgical advice available via the online referral system, and other co-morbidities and co-existing acute medical problems managed by their local medical teams. Decision-making relies on spoke centre teams providing robust and up to date information on the online referral system to allow the neurosurgical team to make management decisions remotely; without the appropriate detail and ability to review patients in person this has the potential to lack nuance in more complex patients. In our centre, a geriatric trauma team consisting of specialist trauma geriatricians works alongside the Neurosurgical team to provide shared care for older neurotrauma patients with medical needs.

Traditionally cSDH was treated either neurosurgically via burr hole trephination or craniotomy or conservatively. In recent years, care of cSDH has evolved beyond conventional neurosurgical intervention. Middle Meningeal Artery Embolisation (MMAE) has emerged as a safe and effective treatment as both a standalone intervention and as an adjunct to surgery [9,10] with the aim of reducing rates of primary or repeat surgery respectively. Case selection criteria for MMAE are still evolving. It has been suggested that MMAE may have substantial value in patients who are at increased risk of progressing to surgical rescue or in those at higher risk of post-surgical recurrence [9,11,12,13].

With modern therapeutic interventions extending beyond conventional neurosurgery, and the complexity that multimorbidity and frailty bring to patients with cSDH, expert and nuanced multi-disciplinary decision-making is required to allow patients to get the right care at the right time. Furthermore, it is important that equitable care is available to all patients regardless of their geographical area, relative to the tertiary neurosurgical centre. It is with this aim that the geriatric trauma team at our centre developed a novel, multidisciplinary cSDH service to work in collaboration with the neurosurgical team to provide advice regarding MMAE suitability and wider cSDH care for older patients across the region. The regional service was initiated through an alteration in the neurosurgical electronic referral pathway and has been reinforced by local meetings and presentations. The aims of this study were to assess service utilisation and activity over time along with impact on regional cSDH patient management. As this was a novel service, and to the authors’ knowledge the first of its kind in the UK, there was no direct comparison to be made. The process of ongoing evaluation began at the inception of the service.

## 2. Methods

We performed a clinical service evaluation of the scope, activity and impact of the new cSDH service through the assessment of referrals that were received by the NBT geriatric trauma team and subsequent patient management over a 21-month period between July 2024 and March 2026.

The study was approved by the institutional audit and clinical effectiveness committee (Ref CAE/QI-489) and did not require formal NHS Research Ethics Committee review in accordance with Health Research Authority guidance.

The evaluation included review of email referrals and responses, records made on a formal electronic referral system (Referapatient.org) and inpatient hospital notes. Referrals from NBT were only included if referred from a non-neurosurgical team.

### 2.1. Referral Process

Referrals to the new cSDH advice service were received via email to the geriatric trauma team at NBT via a dedicated inbox. This was signposted to by the neurosurgical team in their electronic referral responses if deemed appropriate. This largely included patients (1) who were potential candidates for standalone MMAE, (2) who required assessment/management of acute illness or confounding neurological disorders prior to consideration of surgery, and (3) whose multimorbidity and/or frailty required more complex shared decision-making around the risks and benefits of surgery. Referrals were triaged by a geriatric trauma consultant, and if necessary, a follow-up phone call was made to the referring team if more information was needed. Relevant cases were then discussed with Interventional Neuroradiology and Neurosurgery to allow robust multi-disciplinary decision-making. Advice was then conveyed back to the referring team via either email or phone. For documentation and visibility purposes this advice was formally recorded on the electronic referral system.

### 2.2. Data Collection

Demographic data collected included age, gender, clinical frailty score (CFS) and referral source. Outcome data collected included advice type, whether management was enhanced, intervention received, whether conveyed to NBT and mortality. Advice type was divided into single vs. multiple types and then subdivided by advice category. Advice sub-categories were; MMAE suitability, burr hole suitability, antithrombotic management, relevance/clarification of symptoms, prognostication, follow-up scanning, and other. Enhancement of management was defined as a definitive management decision that resulted in either an action or an active decision not to act. Mortality data was collected at 1-, 3-and 6-month time points. The data collected was then analysed using descriptive statistics to assess outcomes and trends.

## 3. Results

### 3.1. Number of Referrals Received

**Figure 1:**
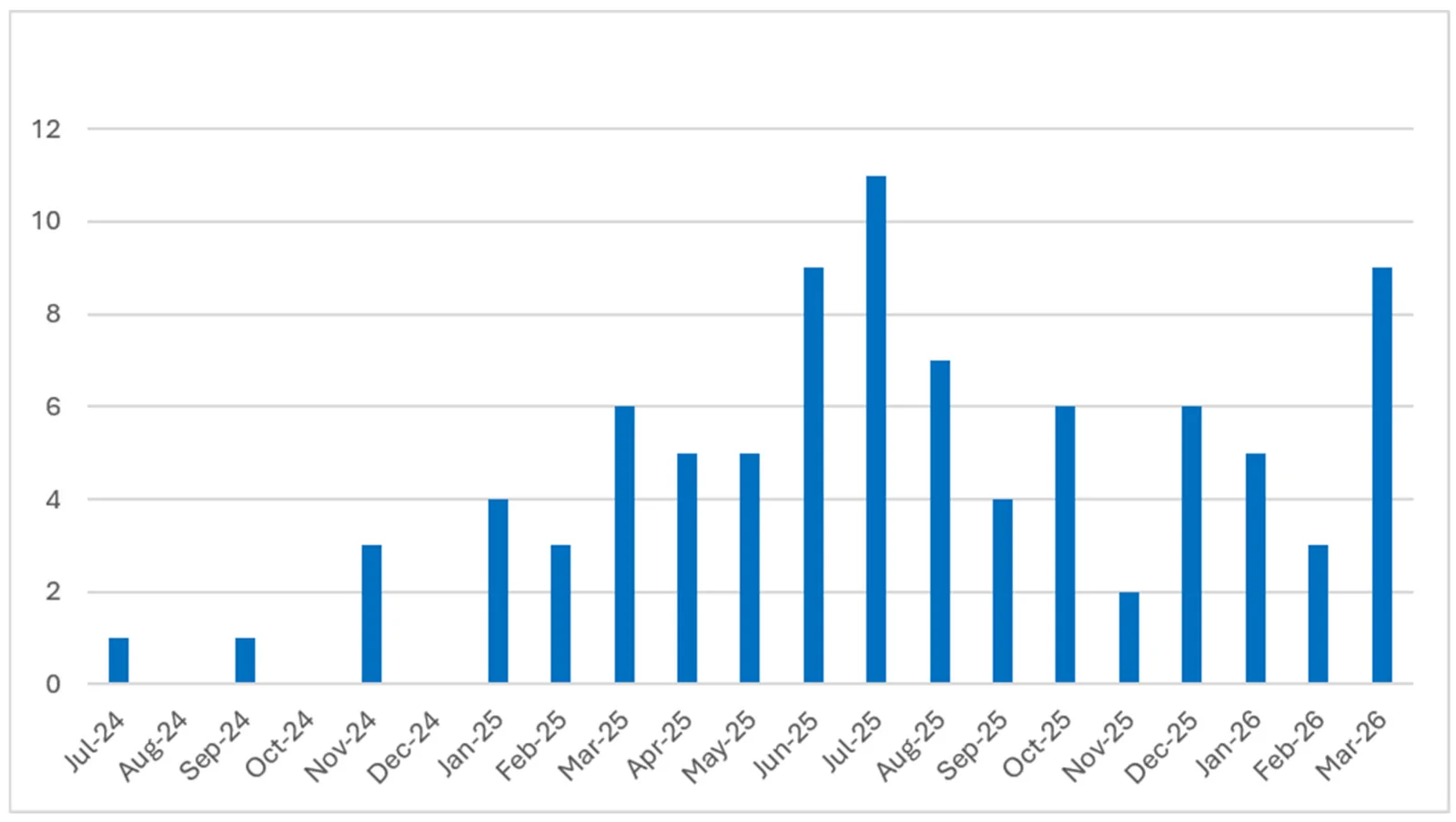
Number of referrals received per month since service inception.

Over the 21-month service evaluation period 92 referrals were received. Two referrals were excluded as the patients were transferred to NBT under neurosurgery prior to geriatric trauma team advice being given, therefore geriatric trauma team input would fall under the shared care agreement for neurosurgical patients. The remaining 90 referrals were included in the service evaluation. The number of referrals received per month is depicted in Figure 1.

### 3.2. Demographic Data

The median age of referred patients was 83 years. The median CFS was 4 with 48.9% of patients defined as non-frail (CFS 1–3), 50% defined as frail (CFS 4–8) and 1.1% defined as terminally ill (CFS 9, in this case haematological malignancy). Only five patients were <65 years old. Whilst strictly speaking CFS is validated for people aged 65 and over, they were assigned a CFS based on their functional status—the majority of these younger patients had no frailty (CFS 1–3).

Referrals were received from all five satellite NHS trusts supported by our tertiary neurosurgical centre. There were also a small number of referrals from general practice and one from a local psychiatric hospital. A variation in referral source constituted a successful outcome as it demonstrated that geographic inequity had been addressed by offering this service to patients beyond our own hospital. Full details can be found in Table 1.

### 3.3. Outcome Measures

Three quarters of the referral responses included multiple advice types. Advice subtypes identified were: MMAE suitability, burr hole suitability, antithrombotic management, relevance/clarification of symptoms, prognostication, follow-up scanning, and other. Advice included in the “other category” included flying advice, Driver and Vehicle Licencing Agency (DVLA) advice, and antiepileptic advice. MMAE suitability was by far the most common type of advice given (93.3%) followed by antithrombotic management advice (51.1%). Full details can be found in Table 2.

Ninety-seven percent of referrals resulted in enhanced management advice. Forty percent of referrals resulted in conversion from conservative management to intervention, with half of these receiving standalone MMAE and half receiving burr holes, either in conjunction with MMAE or standalone. Of all DGH patients referred, 40.3% were conveyed to NBT for intervention. The demographic data for patients converted from conservative management to intervention showed a median age of 83 years (range 59–92 years) and a median clinical frailty score of 3 (range 2–9). Only one of these patients was <65 years old.

Mortality at 1 month was 1.1%, increasing to 6.7% at 3 months and 14.3% at 6 months. At the time of data collection, 1-month and 3-month time points had been passed for all 90 patients and the 6-month time point had come to pass for 77 of the 90 patients.

## 4. Discussion

The clinical parallels of cSDH with fragility fractures and the need for a similar coordinated multidisciplinary approach with the aim of improving outcomes in cSDH patients has previously been emphasised [14]. Frailty has been shown to increase mortality in both conservatively and surgically managed groups, hence the importance of frailty in understanding prognosis and outcomes in cSDH and the drive for early geriatrician input [7]. Recent guidelines highlight the need for an early comprehensive geriatric assessment of these patients [15]. With new evidence-based therapies such as MMAE [11,12,13] (that may, for example, significantly reduce the need for eventual surgery in the older people with frailty), the role of the trauma geriatrician as a gatekeeper is perhaps even more important. To this end, this service evaluation demonstrates that a virtual regional geriatric trauma advice service for cSDH is both feasible and associated with alterations to patient management. Enhanced management advice was observed in almost all patients, potentially indicating that the service may improve access to treatment and address unmet clinical need. The geographic reach of the service spans the catchment areas of five satellite NHS trusts, with 59.7% of DGH patients receiving specialist MDT shared decision-making without requiring transfer to the tertiary centre. This demonstrates the potential for tertiary geriatric trauma services to address regional inequity of access whilst preventing unnecessary patient movement.

The number of referrals received per month increased initially to a peak in July 2025. This initial increase, the authors hypothesise, is due to increasing awareness from referring clinicians of the existence of the novel service. No specific reason other than natural variation was identified for the peak in referrals in July 2025 or the fluctuation thereafter.

The demographic profile of our referral population is consistent with the wider cSDH literature, with more than half of patients displaying frailty [16]. This reflects the typical cSDH patient who is often deemed unsuitable for immediate surgical intervention. This patient cohort may reflect the group in whom specialist geriatrician input is likely to be the most valuable: older adults with multimorbidity, frailty, and antithrombotic use, for whom standard neurosurgical management frameworks may be insufficient to guide nuanced, individualised, shared decision-making.

While assessment of suitability for intervention was the most frequent category of intervention, a significant proportion of referrals requested additional advice on antithrombotic management, symptom interpretation, and prognostication. This reflects the extent to which cSDH management extends beyond purely surgical decision-making and illustrates the importance of collaborative working between neurosurgery and specialist trauma geriatricians.

The 40% conversion rate from conservative to interventional management, with MMAE the most common intervention, is significant and demonstrates a successful outcome as crossover to active management occurred as a consequence of our MDT review. This result contrasts existing UK observational cohort data, in which only 3% of conservatively managed patients crossed over to surgical intervention under a symptom-based watch-and-wait model. In this setting, in which there was neither specialist trauma geriatrician input nor access to MMAE, two-year mortality exceeded 50%—worse than hip fracture [7]. The authors explicitly called for early geriatrician involvement and an MDT approach; our service provides a practical model for delivering collaborative care for a complex pathology that neither specialty is optimally placed to manage alone. By combining neurosurgical expertise in cranial pathology and procedural decision-making with geriatrician expertise of frailty, multimorbidity, and antithrombotic use, together the MDT can identify patients suitable for intervention who might otherwise be managed conservatively. For example, we have previously demonstrated that in a population of cSDH patients with high incidence of frailty and antithrombotic use, judicious use of MMAE resulted in a significant reduction in the rate of treatment failure despite earlier reintroduction of antithrombotic medication [9].

Existing data indicate that cSDH may represent a marker of frailty rather than its cause; whether a multi-specialist management approach translates into meaningful functional benefit remains an important question for future research. Importantly, this system of referral also provides a model in which regional MMAE may be delivered to patients who do not require neurosurgery across the whole network, thus improving equitable access.

Our mortality data show a 1-month mortality rate of only 1.1%, rising to 14.3% at 6 months. Existing UK data on conservatively managed cSDH have shown considerably higher mortality at these time points, with rates of up to 35% at 6 months [7]; by comparison, our cohort shows an absolute mortality reduction of 14–22 percentage points across the three time points [7]. As the comparator data are age-adjusted rather than crude, and our cohort’s age distribution has not been formally matched against theirs, this comparison should be interpreted with some caution. Nonetheless, the magnitude of the difference may suggest that MDT input, including specialist trauma geriatrician assessment, contributes to improved mortality outcomes. The impact of geriatrician input on outcomes for neurosurgical patients in the UK remains an unstudied area—neurosurgery is one of the less-researched fields of perioperative care for older people compared with, for example, gastrointestinal surgery and major trauma, possibly reflecting the absence of organisational and financial incentives available in other specialties, such as the best practice tariff for emergency laparotomy and the National Hip Fracture Database. Further dedicated research into the outcomes of geriatrician input in neurosurgical care is needed.

There are however several limitations to this study. As a single-centre service evaluation, the findings may not be generalisable to other neurosurgical networks with different configurations, populations and resources. No data regarding socio-economic status were collected and would perhaps have provided a greater insight into the population catered for by this novel service. Furthermore, our referral pathway is dependent on the neurosurgical team identifying patients who would benefit from geriatrician input. Indeed, it is worth highlighting that a total of 755 cSDH referrals were made to the neurosurgical electronic referral system over the study timeframe, meaning that our referral service received 11.9% of the total number of cSDH referrals. This has implications in terms of selection bias likely impacting our results and highlights the need for ongoing research to better define the subset of cSDH patients who benefit from enhanced MDT decision-making and thus the development of clearer referral criteria; we envisage that there will be a greater proportion of patients with frailty and multimorbidity who could benefit from such an enhanced MDT service. Although data regarding age and frailty score were collected, more specific clinical information for patients converted from conservative management to intervention such as clinical deficits and midline shift could have provided more clarity on which patients are benefitting the most from this service. Moreover, outcome data beyond immediate management enhancement and mortality have not been captured in this initial short report. Prospective studies, ideally including longer term outcome measures, focusing on patient-reported outcomes, clinician-reported satisfaction, functional status, and health economic analysis, will be required to fully evaluate the value and cost-effectiveness of this model.

## 5. Conclusions

We have demonstrated that a regional geriatric trauma team service is a feasible intervention to provide remote specialist input alongside Neurosurgery to older people with cSDH across a single UK neurosurgical network. Specialist trauma geriatrician review was associated with enhanced management advice in 97.8% of cases and led to therapeutic intervention in 40% of patients who had initially been managed conservatively. Our data also indicates that specialist trauma geriatrician input in the management of neurosurgical patients may improve mortality. Further research is needed to better define the subset of cSDH patients who might benefit from enhanced MDT decision-making, as well as more in-depth evaluation as to whether such models of care improve longer term outcomes for older patients with cSDH.

## Figures and Tables

**Table 1:** Baseline demographics of referral population.

Baseline Characteristic	Number (%)
**Age**	
Median [IQR]	83 [77–88]
Range	50–93
**Gender**	
Male	63 (70)
Female	27 (30)
**Clinical Frailty Scale**	
Median	4
No frailty (CFS 1–3)	44 (48.9)
Very mild—mild frailty (CFS 4–5)	24 (26.7)
Moderate frailty (CFS 6)	11 (12.2)
Severe—very severe frailty (CFS 7–8)	10 (11.1)
Terminally ill (CFS 9)	1 (1.1)
**Referral Source**	
North Bristol NHS Trust	18 (20)
University Hospitals Bristol and Weston NHS Foundation Trust	16 (17.8)
Royal United Hospital Bath NHS Trust	12 (13.3)
Gloucestershire NHS Trust	19 (21.1)
Somerset NHS Trust	21 (23.3)
General practice	3 (3.3)
Psychiatric hospital	1 (1.1)

**Table 2:** Outcome data including advice type and management alteration.

Outcome	Number (%)
**Advice Type**	
Single	22 (24.4)
Multiple	68 (75.6)
**Advice Subtypes (Note That More Than One Sub-Type Can Apply)**	
MMAE suitability	84 (93.3)
Burr hole suitability	19 (21.1)
Antithrombotic management advice	46 (51.1)
Relevance/clarification of symptoms	12 (13.3)
Prognostication	4 (4.4)
Repeat scanning	21 (23.3)
Other	6 (6.7)
Flying advice	3
Antiepileptic advice	2
DVLA advice	1
**Enhancement in Management**	
Yes	88 (97.8)
No	2 (2.2)
**Conversion From Initial Conservative Management to Intervention**	
Total	36 (40)
**Intervention Received**	
Standalone MMAE	18 (50)
Standalone burr holes	7 (19.4)
Burr holes and adjunctive MMAE	11 (30.6)
**Conveyed to NBT for Intervention if DGH Patient (Total DGH Patients n = 72)**	
Yes	29 (40.3)
No	43 (59.7)
**Mortality**	
At 1 month	1 (1.1)
At 3 months	6 (6.7)
At 6 months	11 (14.3)

## Data Availability

The original contributions presented in this study are included in the article. Further inquiries can be directed to the corresponding authors.
